# Papillary thyroid carcinoma with hyperthyroidism and multiple metastases: A case report

**DOI:** 10.1097/MD.0000000000021346

**Published:** 2020-07-24

**Authors:** Li-li Zhang, Bin Liu, Fang-fang Sun, Hong-yu Li, Shuang Li, Li-rong Zhao

**Affiliations:** a Department of Ultrasound; b Department of Hand Surgery, The First Hospital of Jilin University, Changchun, China.

**Keywords:** hyperthyroidism, metastases, papillary thyroid carcinoma, skeletal muscles, thyroid cancer

## Abstract

**Rationale:**

Papillary thyroid carcinoma (PTC) is the most common type of primary thyroid cancer with a low incidence of distant metastases. PTC represents more than 70% to –90% of thyroid malignancies. Distant metastases have only been observed in only 1% to 15% of patients with PTC. In this article, we reported the case of a patient with PTC and hyperthyroidism as well as simultaneous multiple metastases.

**Patient concerns:**

A 47-year-old man was admitted to our hospital on February 22, 2019, with several neck masses that had been present for 12 months, low back pain for 9 months, and lower limb paraplegia for 3 months.

**Diagnoses:**

According to the patient physical examination, adjuvant examination (e.g., ultrasound, computed tomography, magnetic resonance imaging, blood test, and biopsy) and medical history, the clinical diagnosis was as follows: thyroid papillary carcinoma; cervical lymph node metastasis; multisite bone metastasis (6th and 7th cervical vertebrae, left clavicle proximal, right scapula bone, thoracic vertebrae, lumbar vertebrae, sacral vertebrae, bilateral ilium, and left pubic bone); muscular metastasis (the right medial femoral muscle, the vastus lateralis muscle, left thigh muscle, and the flexor superficialis of the left forearm); possible mediastinal lymph node metastasis; and paraplegia due to the soft-tissue metastasis around the 9th thoracic vertebral spine; and hyperthyroidism (free thyroxine: 36.59 pmol/L, free triiodothyronine: 9.58 pmol/L, thyroid-stimulating hormone: 0.005 μIU/mL, thyroid autoantibody: 2.53 IU/L).

**Interventions and outcomes:**

The patient refused to undergo further intervention or follow-up.

**Lessons:**

In summary, this is the 1st case of in which a patient with PTC and hyperthyroidism, as well as simultaneous multiple skeletal muscles and bone metastases, lymph node metastasis, and paraplegia was observed. In practice, in cases where patients have PTC and hyperthyroidism, practitioners should perform further examinations to rule out the presence of distant metastases. We believe that the use of ultrasound has a unique advantage in the diagnosis of PTC and skeletal muscle metastasis.

## 1 Introduction

Papillary thyroid carcinoma (PTC) is the most common type of primary thyroid cancer. This form of carcinoma represents more than 70% to 90% of thyroid malignancies.^[1]^ PTC is a slowly progressive cancer with high survival rate.^[2,3]^ Distant metastases have only been observed in only 1% to 15% of patients with PTC. Metastases are most observed in the lungs, followed by the bones.^[4,5]^ Muscular and soft-tissue metastases of PTC are very rare.

In this article, we reported the case of a patient with PTC and hyperthyroidism as well as simultaneous multiple skeletal muscles and bone metastases, lymph node metastasis, and paraplegia. We also present a literature review, aiming to provide further evidence regarding the diagnosis, intervention, and prognosis of PTC with distant metastasis.

## 2 Case report

### 2.1 Patient information

A 47-year-old man was admitted to the First Hospital of Jilin University on February 22, 2019, with several neck masses that had been present for 12 months. The patient had also experienced low back pain for 9 months and lower limb paraplegia for 3 months. The patient had no family history of thyroid disease or any history of exposure to external or accidental radiation.

### 2.2 Clinical findings

Physical examination of the patient revealed the presence of multiple masses in the area of the right supraclavicular fossa and the right neck. The largest lump, of approximately 2 × 2 cm, was hard, mobile, and painless. Three additional lumps were identified in the left forearm, the anterior side of the right thigh, and the lateral side of the right thigh. These lumps were 3 × 2 cm, 3 × 2 cm, and 2 × 2 cm, respectively. All 3 lumps were hard, solid, solitary, and tender. No sensation or pain was observed below the line of the anterior superior iliac spine, and the lower extremities were paraplegic.

Blood tests for thyroid function revealed that the free thyroxine level was 36.59 pmol/L (reference value 11.50–22.70 pmol/L), the free triiodothyronine level was 9.58 pmol/L (reference value 3.50–6.50 pmol/L), the thyroid-stimulating hormone (TSH) level was 0.005 μIU/mL (reference value 0.550–4.780 μIU/mL), and the thyroid autoantibody (TRAb) level was 2.53 IU/L (reference value 0.30–1.22 IU/L). No abnormalities were observed in the expression of tumor markers.

**Figure 1:**
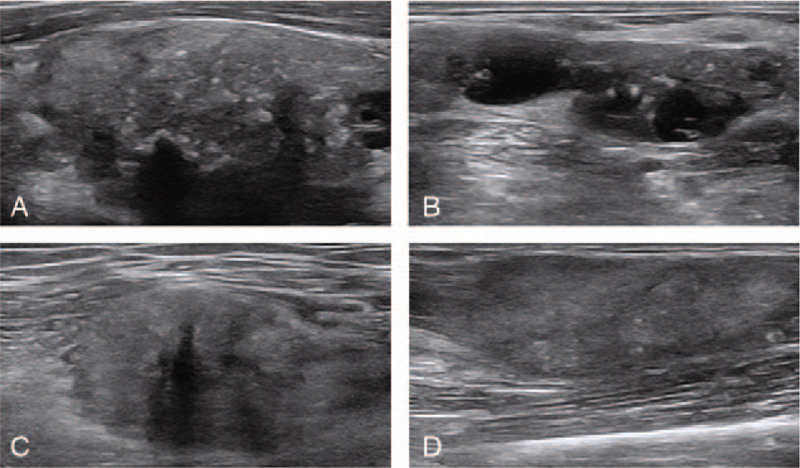
Ultrasound images. (A) A hypoechoic nodule with blurred borders can be seen on the upper right thyroid gland, and punctate calcification can be seen inside. (B) The right cervical lymph node has lost its hilum structure. There are solid hyperechoic and cystic areas in the lymph nodes. (C) A hypoechoic nodule can be seen in the middle part of the right thigh femoris, where punctate calcification is present. (D) A hypoechoic nodule can be seen in the middle part of the superficial flexor of the left forearm.

Ultrasound examination of the thyroid and neck revealed a 4.2 × 2.6 cm hypoechoic nodule with blurred borders, an irregular shape, and uneven and visible internal scattered punctate hyperechoic signs on the right lobe of the thyroid gland (Fig. 1A). A rich blood flow signal was detected by Doppler ultrasound. Another similar nodule, 1.5 × 0.9 cm in size, was observed in the thyroid isthmus. Multiple malformed lymph nodes (levels III and IV) were observed in the right neck, in which the structure of the hilum was unclear. There were solid hyperechoic and cystic areas in the lymph nodes and multiple punctate hyperechoic signs in the solid components of the nodules. The size of the larger lymph node was 2.9 × 2.3 cm (Fig. 1B). Ultrasound examination of the right thigh revealed a 2.4 × 2.1 cm substantial hypoechoic mass in the middle of the medial femoral muscle with a blurred boundary, irregular shape, uneven internal echo, and multiple punctate hyperechoic signs (Fig. 1C). A rich blood flow signal was detected by Doppler ultrasound. Two additional lumps of the same nature were observed in the middle of the vastus lateralis muscle and the flexor superficialis of the left forearm, with sizes of 3.1 × 1.6 cm and 4.0 × 1.5 cm, respectively (Fig. 1D). The ultrasound findings were as follows: hypoechoic thyroid nodules with calcification, considered to indicate thyroid cancer; cervical lymphadenopathy with calcification, considered to indicate lymph node metastasis; right thigh and left forearm muscle tissue lump with calcification without excluding to thyroid cancer metastasis.

**Figure 2:**
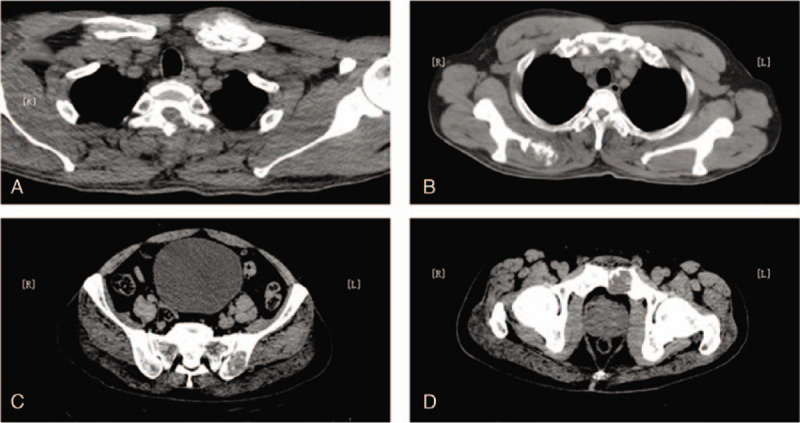
Computed tomography images of multisite bone destruction. (A) Left clavicle bone destruction; (B) right scapula bone destruction; (C) left iliac bone destruction; (D) left pubic bone destruction.

Computed tomography (CT) scan results revealed multiple points of bone destruction in the 6th and 7th cervical vertebrae, left clavicle proximal, right scapula bone, thoracic vertebrae, lumbar vertebrae, sacral vertebrae, bilateral ilium, and left pubic bone (Fig. 2). The 9th thoracic vertebrae had obvious bone destruction, the spinal canal was destroyed, and the imaging of the spinal cord revealed a blurred structure. Pulmonary CT revealed a slight inflammation of the left lower lobe and enlarged mediastinal lymph nodes. The CT findings were as follows: multiple site bone destruction, not excluding metastatic cancer, and mediastinal lymphadenopathy, not excluding metastatic cancer.

**Figure 3:**
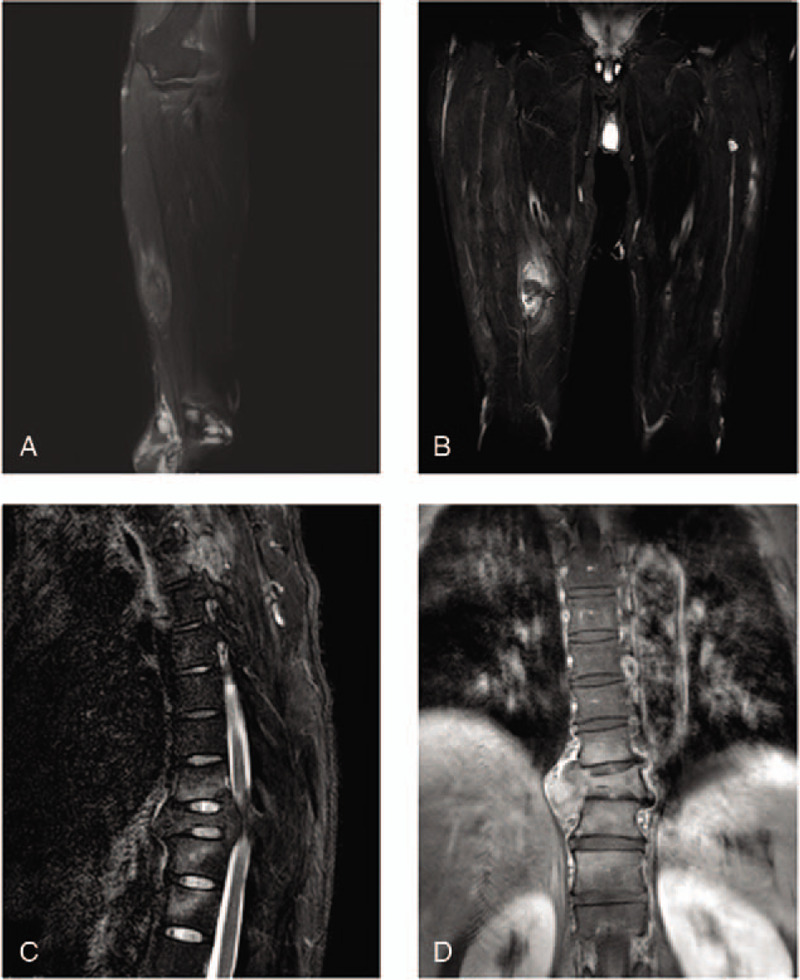
Magnetic resonance images. (A) A lump can be seen on the ulnar side of the left forearm muscle with a slightly higher signal in the T2-fat-suppression images. (B) A lump can be seen in the middle part of the right thigh with an equal signal in fat-suppression images, and a high signal in the peripheral muscle tissue. (C) The body of the 9th thoracic vertebrae is flattened and wedge shaped. There is a high patchy fat-suppression signal in the spinal cord. (D) A strip-like abnormal signal and mild uneven enhancement in the soft tissue around the 9th thoracic vertebrae can be observed in an enhanced scan.

Magnetic resonance imaging (MRI) revealed a 3.6 × 1.5 cm elliptical abnormal signal in the soft tissue of the left forearm ulnar with equal signal intensity on T1-weighted images (T1WI) and slightly high and low mixed signals on T2-weighted images (T2WI; Fig. 3A). Two other nodular abnormal signals were observed in the right thigh muscle group in the lower middle horizontal part and outer part separately with equal and slightly lower signals on T1WI and equal signals on fat-suppression images. A number of small mass signals were observed in the left thigh muscle group, with equal signal on the T1 image (Fig. 3B). Thoracic MRI with enhanced scanning revealed multiple thoracic vertebral bodies with multiple patchy abnormal signals, and an enhanced scan revealed mild uneven enhancement. The body of 9th Thoracic Vertebra was wedge shaped with a high patchy fat-suppression signal in the spinal cord. In some of the soft tissues around the thoracic vertebral body, strip-like abnormal signals were observed with low signal on T1WI, and high and low mixed signals on T2WI; enhanced scanning revealed mild uneven enhancement (Fig. 3C and D). The MRI findings were as follows: a muscle mass occupying lesion was observed in the left forearm and bilateral thigh; there was horizontal spinal cord compression at the 9th thoracic vertebral body and edema due to the soft-tissue metastasis around the spine, and the 9th thoracic vertebral compression fracture.

**Figure 4:**
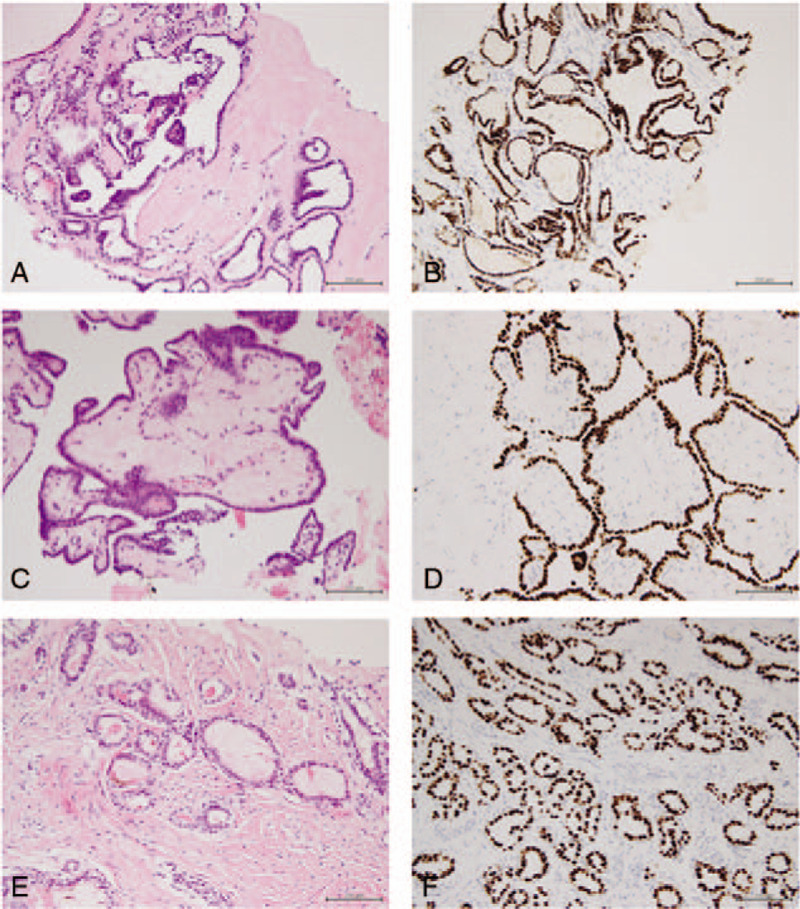
Pathologic slide (hematoxylin and eosin staining) images. (A, B) Thyroid follicular epithelium cells can be seen with atypical, partial papillary hyperplasia, immunohistochemistry TTF-1(+) (×200). (C, D) Papillary carcinoma metastasis can be seen in lymph nodes, immunohistochemistry TTF-1(+) (×200). (E, F) Papillary carcinoma metastasis can be seen in the right thigh lump, immunohistochemistry TTF-1(+) (×200).

The patient underwent an ultrasound-guided biopsy of the thyroid nodules, right cervical lymph nodes, and right thigh mass under local anesthesia on February 26, 2019. The postoperative pathologic report revealed: pleomorphism of the follicular epithelium, partial papillary hyperplasia, and glassy nuclei presence in the thyroid nodule sample with a diagnosis of thyroid papillary carcinoma (Fig. 4A and B); the biopsy tissue in the right cervical lymph node was consistent with thyroid papillary carcinoma metastasis with immunohistochemical results of cytokeratin 19 (CK19) (+), cytokeratin 7 (CK7) (+), thyroglobulin (TG) (weak +), TTF-1(+), cytokeratin 20 (CK20) (−), napsin A (−), and villin (−) (Fig. 4C and D); the biopsy tissue in the right thigh mass was consistent with thyroid papillary carcinoma metastasis with immunohistochemical results of CK19(+), CK7(+), TG(+), TTF-1(+), CK20(−), napsin A(−), and villin(−) (Fig. 4E and F). The patient refused to undergo further examination, such as positron emission tomography-CT (PET-CT), to determine whether additional lymph node or distant metastases were present.

### 2.3 Diagnostic assessment

According to the physical examination, adjuvant examination, and medical history of the patient, the clinical diagnosis was as follows: thyroid papillary carcinoma; cervical lymph node metastasis; multisite bone metastasis (6th and 7th cervical vertebrae, left clavicle proximal, right scapula bone, thoracic vertebrae, lumbar vertebrae, sacral vertebrae, bilateral ilium, and left pubic bone); muscular metastasis (the right medial femoral muscle, the vastus lateralis muscle and the flexor superficialis of the left forearm); possible mediastinal lymph node metastasis; and paraplegia due to soft-tissue metastasis around the 9th thoracic vertebral spine; hyperthyroidism.

### 2.4 Treatment and follow-up

The patient refused to undergo further intervention and asked to be discharged from the hospital on March 20, 2019. He did not consent to take part in any follow-up observations.

## 3 Discussion

The PTC is mainly transferred to the local lymph nodes through lymphatic drainage, and distant metastasis is very rare. However, in addition to the observation of metastasis in the lung and bone, there have been reports of metastasis in very rare sites such as the skeletal muscle (see details in Table 1), brain,^[33]^ breasts,^[34]^ parotid gland,^[35]^ liver,^[36]^ adrenal gland,^[37]^ kidney,^[37]^ ovary,^[38]^ and the nasal cavity.^[39]^ The presence of distant metastasis indicates a poor prognosis. In such cases, the 10-year survival rate is only around 50%.^[40]^

**Table 1:**
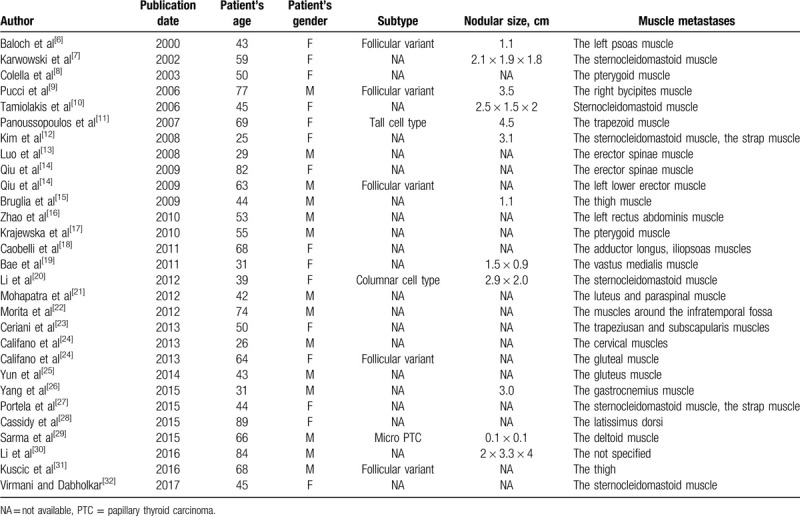
Reported muscle metastases from PTC previously in the literature.

Cervical lymph nodes are the most prone to metastasis in PTC, and 23% to 56% of patients with PTC have clinically significant lymph node metastasis; while in cervical lymph node prophylactic dissection, in up to 90% of cases the histologic results reveal lymph nodes metastasis.^[41]^ Lymph node metastasis is also a risk factor for high recurrence of the disease.^[42,43]^ In the present case, right cervical lymph node metastasis was observed.

The incidence of bone metastasis in PTC has been reported to be around 6.9%.^[44]^ The size of PTC nodules is closely related to the occurrence of bone metastasis, and the risk of bone metastasis for nodules larger than 4 cm is 1.54 times that for nodules smaller than 1 cm.^[45]^ The spine is the most vulnerable site of PTC bone metastasis, and 28% of patients with spinal metastasis will 1st show pain at the corresponding metastatic site.^[46,47]^ In the present case, the patient developed back pain 9 months before admission to hospital. Spinal metastasis often leads to neuronal motor paralysis of the lower extremities, various sensory impairments, defecation disorders, and nerve root pain at the corresponding metastatic site due to compression of the spinal cord.^[48]^ In this case, the mass transfer in the para-thoracic soft tissue of the 9th thoracic vertebra invaded the spinal canal and compressed the spinal cord, which likely resulted in the symptoms of lower back pain, paraplegia of both lower limbs, sensory disorder, and defecation disorders.

Skeletal muscle metastasis of PTC is very rare. Herbowski reviewed the literature and calculated the incidence of skeletal muscle metastases in PTC and follicular thyroid carcinoma (FTC) to be around 4/10.^[36,49]^ There is a hypothesis that skeletal muscle can tolerate lactic acid produced by tumor cells, thereby inhibiting the neovascularization of tumors; in addition, the pH environment in skeletal muscle and the movement of skeletal muscle can inhibit the proliferation of tumor cells.^[50]^ We reviewed the literature from 1999 to 2018 and found that only 29 cases of PTC skeletal muscle metastases were diagnosed, affecting 18 parts of the skeletal muscle (Table 1). Among these 29 cases, we found the most common site of metastases was the sternocleidomastoid muscle (6 cases in total), which included 4 cases of needle biopsy or implant transfer after laparoscopic treatment, and the 2nd common metastatic site was the thigh and buttock muscle (3 cases in total). For the pathologic category, only 5 cases were reported as being of follicular subtypes, and 2 cases were reported as columnar epithelial subtypes. The maximum diameter of PTC nodules with skeletal muscle metastases ranged from 1.1 to 4.5 cm. In the present case, the patient had multisite muscle metastases, including the forearm and thigh muscles. This is the first report of such a case. Another interesting case of skeletal muscle metastasis of PTC was reported by Sarma et al^[29]^ in which the patient had both FTC (3.3 × 2.5 × 2 cm) and micro PTC (0.1 × 0.1 cm). The left deltoid muscle metastasis from micro PTC was found 6 months after total thyroidectomy. It was believed that the FTC was more likely to be the cause of the distant metastasis.^[9,29]^ However, in the case reported by Sarma and colleagues, the metastatic carcinoma of the skeletal muscle came from a small positive micro PTC.^[29]^

In the present case, the blood test results (free thyroxine: 36.59 pmol/L, free triiodothyronine: 9.58 pmol/L, TSH: 0.005 μIU/mL, and TRAb: 2.53 IU/L) indicated that the patient had hyperthyroidism with no systemic treatment. This may have been a factor in the development of multiple bones and skeletal muscle metastases in this patient, as the high blood flow state that occurs during hyperthyroidism may have promoted the spread of tumor cells, making thyroid cancer more prone to distant metastasis. This hypothesis is consistent with the idea of Ito et al.^[43]^

Doppler ultrasound is the 1st choice for the examination of the thyroid and soft-tissue masses due to its economic and convenient advantages. Although the transfer of PTC to skeletal muscle is very rare, it is necessary to consider the possibility of PTC metastasis when encountering a substantial mass of microcalcification in the skeletal muscle. For the overall assessment of PTC distant metastasis, 18-fluorodeoxyglucose PET-CT (^18^F FDG PET-CT) imaging can accurately assess the disease by detecting the local lymph node involvement and distant metastasis.^[24]^ Unfortunately, in this case, the patient refused to undergo PET-CT examination.

In summary, this is the 1st case in which a patient with PTC and hyperthyroidism was shown to have simultaneous multiple skeletal muscle and bone metastases, lymph node metastasis, and paraplegia. The point of interest in the present case is that the high blood flow state of hyperthyroidism may be one of the factors that contributed to the occurrence of multiple distant metastases.

Therefore, in practice, for patients with PTC and hyperthyroidism, practitioners should perform further examinations to rule out the presence of distant metastases. We believe that the use of ultrasound has a unique advantage in the diagnosis of PTC with skeletal muscle metastasis.

## Author contributions

Lili Zhang, Bin Liu, and Lirong Zhao designed the study, conducted all searches, appraised all potential studies and wrote and revised the draft manuscript and subsequent manuscripts. Fangfang Sun and Hongyu Li revised the draft manuscript and subsequent manuscripts. Shuang Li assisted with the presentation of findings and assisted with drafting and revising the manuscript. All authors read and approved the final manuscript.

## References

[1] Jameson JLWA Disorders of the thyroid gland. Harrison's Principles of Internal Medicine 2001;2:2060–83.

[2] La Vecchia C Malvezzi M Bosetti C Thyroid cancer mortality and incidence: a global overview. Int J Cancer 2015;136:2187–95. 25284703 10.1002/ijc.29251

[3] Sebastian SO Gonzalez JMR Paricio PP Papillary thyroid carcinoma: prognostic index for survival including the histological variety. Arch Surg 2000;135:272–7. 10722027 10.1001/archsurg.135.3.272

[4] Mazzaferri EL Massoll N Management of papillary and follicular (differentiated) thyroid cancer: new paradigms using recombinant human thyrotropin. Endocr Relat Cancer 2002;9:227–47. 12542401 10.1677/erc.0.0090227

[5] Ito Y Masuoka H Fukushima M Prognosis and prognostic factors of patients with papillary carcinoma showing distant metastasis at surgery (M1 patients) in Japan. Endocr J 2010;57:523–31. 20379035 10.1507/endocrj.k10e-019

[6] Baloch ZW LiVolsi VA Encapsulated follicular variant of papillary thyroid carcinoma with bone metastases. Mod Pathol 2000;13:861–5. 10955452 10.1038/modpathol.3880153

[7] Karwowski JK Nowels KW McDougall IR Needle track seeding of papillary thyroid carcinoma from fine needle aspiration biopsy. A case report. Acta Cytol 2002;46:591–5. 12040660 10.1159/000326884

[8] Colella G Capone R Cappabianca S Mandibular metastasis from papillary thyroid carcinoma--a case report. Tumori 2003;89:452–4. 14606655 10.1177/030089160308900422

[9] Pucci A Suppo M Lucchesi G Papillary thyroid carcinoma presenting as a solitary soft tissue arm metastasis in an elderly hyperthyroid patient. Case report and review of the literature. Virchows Arch 2006;448:857–61. 16568308 10.1007/s00428-006-0187-4

[10] Tamiolakis D Antoniou C Venizelos J Papillary thyroid carcinoma metastasis most probably due to fine needle aspiration biopsy. A case report. Acta Dermatovenerol Alp Pannonica Adriat 2006;15:169–72. 17982609

[11] Panoussopoulos D Theodoropoulos G Vlahos K Distant solitary skeletal muscle metastasis from papillary thyroid carcinoma. Int Surg 2007;92:226–9. 18050833

[12] Kim JH Choi YJ Kim JA Thyroid cancer that developed around the operative bed and subcutaneous tunnel after endoscopic thyroidectomy via a breast approach. Surg Laparosc Endosc Percutan Tech 2008;18:197–201. 18427342 10.1097/SLE.0b013e318168dda4

[13] Luo Q Luo QY Sheng SW Localization of concomitant metastases to kidney and erector spinae from papillary thyroid carcinoma using (131)I-SPECT and CT. Thyroid 2008;18:663–4. 18578619 10.1089/thy.2007.0326

[14] Qiu ZL Luo QY Erector spinae metastases from differentiated thyroid cancer identified by I-131 SPECT/CT. Clin Nucl Med 2009;34:137–40. 19352273 10.1097/RLU.0b013e31819675b6

[15] Bruglia M Palmonella G Silvetti F Skin and thigh muscle metastasis from papillary thyroid cancer. Singapore Med J 2009;50:e61–4. 19296013

[16] Zhao LX Li L Li FL Rectus abdominis muscle metastasis from papillary thyroid cancer identified by I-131 SPECT/CT. Clin Nucl Med 2010;35:360–1. 20395716 10.1097/RLU.0b013e3181d6265b

[17] Krajewska J Olczyk T Roskosz J Treatment with sorafenib in advanced thyroid cancer - a case report. Endokrynologia Polska 2010;61:492–6. 21049464

[18] Caobelli F Paghera B Panarotto MB Two distant muscular metastases from papillary carcinoma of the thyroid demonstrated by (18)F-FDG PET/CT and confirmed by biopsy. Nucl Med Mol Imaging 2011;45:324–5. 24900025 10.1007/s13139-011-0112-x PMC4043048

[19] Bae SY Lee SK Koo MY Distant, solitary skeletal muscle metastasis in recurrent papillary thyroid carcinoma. Thyroid 2011;21:1027–31. 21834676 10.1089/thy.2010.0249

[20] Li S Zhang F Zhang Y Implantation at sternocleidomastoid and chest wall after endoscopic thyroid carcinoma surgery. Surg Laparosc Endosc Percutan Tech 2012;22:e239–42. 22874712 10.1097/SLE.0b013e318259f43b

[21] Mohapatra T Arora A Bethune NN Coexisting iodine avid and iodine nonconcentrating lesions with multiple distant soft tissue metastasis in papillary thyroid cancer. Indian J Nucl Med 2012;27:38–41. 23599598 10.4103/0972-3919.108853 PMC3628261

[22] Morita N Morimoto K Yonezawa K Infratemporal fossa metastasis of papillary thyroid cancer. Head Neck 2012;35:E119–21. 22495817 10.1002/hed.21951

[23] Ceriani L Paone G Giovanella L Unusual muscular metastases from papillary thyroid carcinoma detected by fluorine-18-fluorodeoxyglucose PET/MRI. J Clin Endocrinol Metab 2013;98:2208–9. 23596135 10.1210/jc.2013-1472

[24] Califano I Quildrian S Coduti M Soft tissue metastases from differentiated thyroid cancer diagnosed by 18F FDG PET-CT. Arq Bras Endocrinol Metabol 2013;57:317–21. 23828437 10.1590/s0004-27302013000400007

[25] Yun KJ Kim W Kim EH Accelerated disease progression after discontinuation of sorafenib in a patient with metastatic papillary thyroid cancer. Endocrinol Metab (Seoul) 2014;29:388–93. 25309799 10.3803/EnM.2014.29.3.388 PMC4192805

[26] Yang J Li LF Zhang XM Unusual synchronous skeletal muscle and lung metastasis in papillary thyroid cancer: a case report and review of the literature. Oncol Lett 2015;9:727–30. 25621043 10.3892/ol.2014.2742 PMC4301470

[27] Portela RA Choby GW Manni A Unusual sites of metastasis of papillary thyroid cancer: case series and review of the literature. Ear Nose Throat J 2015;94:E43–7. 26322457

[28] Cassidy D Gupta G Graham R Metastasis of papillary thyroid cancer to the soft tissue of the back in the setting of recurrent disease. Am Med Student Res J 2015;1:206–11.

[29] Sarma M Sonik B Subramanyam P Isolated skeletal muscle metastatic deposit in a patient with micropapillary carcinoma thyroid identified by 18F FDG PET CT. J Egypt Natl Canc Inst 2015;27:47–50. 25698468 10.1016/j.jnci.2015.01.002

[30] Li ZG Lin ZC Mu HY Polysplenia syndrome with splenic and skeletal muscle metastases from thyroid carcinoma evaluated by FDG PET/CT: case report and literature review: a care-compliant article. Medicine 2016;95:e2532. 26825891 10.1097/MD.0000000000002532 PMC5291561

[31] Kuscic LJ Klancnik M Paladin I Obstructive nephropathy caused by renal metastasis of papillary thyroid carcinoma: a case report. Endocr Oncol Metab 2016;2:94–8.

[32] Virmani N Dabholkar J Distant skeletal muscle metastasis to sternocleidomastoid in the setting of recurrent papillary thyroid carcinoma. Thyroid Res Practice 2017;14:77–80.

[33] Martin Martin G Ismail A Sancho Moya C Brain metastases as the first clinical sign of papillary thyroid cancer [in Spanish]. Cir Esp 2011;89:472–4. 21470600 10.1016/j.ciresp.2010.04.017

[34] Loureiro MM Leite VH Boavida JM An unusual case of papillary carcinoma of the thyroid with cutaneous and breast metastases only. Eur J Endocrinol 1997;137:267–9. 9330591 10.1530/eje.0.1370267

[35] Markitziu A Fisher D Marmary Y Thyroid papillary carcinoma presenting as jaw and parotid gland metastases. Int J Oral Maxillofac Surg 1986;15:648–53. 3097195 10.1016/s0300-9785(86)80074-6

[36] Nimmagadda A Krishna Mohan VS Manthri R Unusual metastases in papillary microcarcinoma of thyroid. Indian J Nucl Med 2019;34:32–4. 30713376 10.4103/ijnm.IJNM_127_18 PMC6352647

[37] Ginzburg S Reddy M Veloski C Papillary thyroid carcinoma metastases presenting as ipsilateral adrenal mass and renal cyst. Urol Case Rep 2015;3:221–2. 26793559 10.1016/j.eucr.2015.08.007 PMC4714304

[38] Pirvu A Guigard S Blaise H Peroperative detection with a gamma probe of pelvic metastasis after differentiated thyroid carcinoma in female patients: about two cases and management reflections. Chirurgia (Bucharest, Romania: 1990) 2013;108:126–9. 23464784

[39] Pourseirafi S Shishehgar M Ashraf MJ Papillary carcinoma of thyroid with nasal cavity metastases: a case report. Iran J Med Sci 2018;43:90–3. 29398758 PMC5776001

[40] Elisei R Molinaro E Agate L Are the clinical and pathological features of differentiated thyroid carcinoma really changed over the last 35 years? Study on 4187 patients from a single Italian institution to answer this question. J Clin Endocrinol Metab 2010;95:1516–27. 20156922 10.1210/jc.2009-1536

[41] Caron NR Clark OH Papillary thyroid cancer surgical management of lymph node metastases. Curr Treat Options Oncol 2005;6:311–22. 15967084 10.1007/s11864-005-0035-9

[42] Baek SK Jung KY Kang SM Clinical risk factors associated with cervical lymph node recurrence in papillary thyroid carcinoma. Thyroid 2010;20:147–52. 19785522 10.1089/thy.2008.0243

[43] Ito Y Kihara M Takamura Y Prognosis and prognostic factors of papillary thyroid carcinoma in patients under 20 years. Endocr J 2012;59:539–45. 22472193 10.1507/endocrj.ej12-0086

[44] Choksi P Papaleontiou M Guo C Skeletal complications and mortality in thyroid cancer: a population-based study. J Clin Endocrinol Metab 2017;102:1254–60. 28324052 10.1210/jc.2016-3906 PMC5460727

[45] Slook O Levy S Slutzky-Shraga I Long-term outcomes and prognostic factors in patients with differentiated thyroid carcinoma and bone metastases. Endocr Pract 2019;25:427–37. 30657361 10.4158/EP-2018-0465

[46] Wexler JA Approach to the thyroid cancer patient with bone metastases. J Clin Endocrinol Metab 2011;96:2296–307. 21816796 10.1210/jc.2010-1996

[47] Kushchayeva YS Kushchayev SV Carroll NM Spinal metastases due to thyroid carcinoma: an analysis of 202 patients. Thyroid 2014;24:1488–500. 24921429 10.1089/thy.2013.0633

[48] Ramadan S Ugas MA Berwick RJ Spinal metastasis in thyroid cancer. Head Neck Oncol 2012;4:39. 22730910 10.1186/1758-3284-4-39 PMC3466148

[49] Herbowski L Skeletal muscle metastases from papillary and follicular thyroid carcinomas: an extensive review of the literature. Oncol Lett 2018;15:7083–9. 29731874 10.3892/ol.2018.8216 PMC5920897

[50] Seely S Possible reasons for the high resistance of muscle to cancer. Med Hypotheses 1980;6:133–7. 7393016 10.1016/0306-9877(80)90079-1

